# Delayed treatment of neglected open knee dislocation; case report

**DOI:** 10.1016/j.ijscr.2022.106937

**Published:** 2022-03-12

**Authors:** Hassan Salad Ibrahim, Engin Ilker Çiçek, Hüseyin Taşkoparan, Abdullahi yusuf Duran Hashi

**Affiliations:** a Mogadishu Somali Turkey Research and Training Hospital, Mogadishu, Somalia; b Jazeera University, Somalia

**Keywords:** Open knee dislocation, Delayed treatment, Gastrocnemius flap, Arthrodesis, Somalia

## Abstract

**Introduction and importance:**

Traumatic open knee dislocation is a rare, severe injury characterized by severe ligamentous destruction and a high frequency of infection and neurovascular involvement. Delayed treatment of these injuries is complicated, necessitating the intervention of not only a skilled orthopedic surgeon but also a plastic surgeon. To the best of our knowledge, this is the first case of delayed open knee dislocation faced by a practicing surgeon in an underdeveloped country (Somalia) with a successful outcome.

**Case presentation:**

A 60 years old diabetic man, presented to our emergency unit with an open wound of his left knee, due to a traffic accident three months ago. At the time, a bone healer reduced the dislocation and applied traditional medicine to cover the wound. We decided to treat the patient with vigorous debridement, gastrocnemius flap, and hybrid external fixation for arthrodesis. The patient was followed up for three months after the surgery with excellent clinical and radiological outcomes.

**Conclusion:**

Although treating delayed open knee dislocation injuries is challenging, using a gastrocnemius muscle flap to cover the knee joint and arthrodesis to stabilize the joint will not only prevent limb amputation but will also result in satisfactory results.

## Highlights

• Traumatic open knee dislocation is a rare, severe injury characterized by ligamentous destruction, high frequency of infection and neurovascular involvement
• Delayed treatment of open knee dislocations is a relatively unusual injury that can be limb threatening
• The use of a gastrocnemius muscle flap and joint arthrodesis to treat these injuries has proven to be beneficial.

## 1 Introduction and importance

Traumatic knee dislocation is a rare, severe injury that causes substantial ligament damage and a high rate of vascular and neurological involvement. Open dislocation of the knee is an uncommon event with uncertain incidence and prognosis, resulting in poor knee joint function at best and above-knee amputation at worst [1]. Neglected open knee dislocation is an even rarer occurrence, and the treatment of these injuries, due to their rarity, is not clearly described in the literature, ranging from joint arthroplasty, arthrodesis, or amputation in extreme cases [2], [3], [4].

Delayed treatment of these injuries can lead to wound problems and ligament reconstruction failure due to the increased risk of infection. In an underdeveloped country where health-care access is difficult, the results are not always satisfactory. We present a case of a three-month-old neglected open knee dislocation treated with a medial gastrocnemius flap and stabilization with a hybrid external fixation in a low-resource nation (Somalia). This case report has been reported in line with the SCARE Criteria [5].

## 2 Case presentation

A 60 years old diabetic man, presented to our emergency unit with an open wound of his left knee, resulting from a traffic accident three months ago causing an open left knee dislocation. At the time, a bone healer reduced the dislocation and applied traditional medicine to cover the wound. There was no history of psychiatric illness or drug use.

Physical examination revealed severe soft tissue loss of the anterior knee with exposed lateral femoral condyle, the extensor mechanism was damaged and the wound was contaminated with particles of traditional medicine. The patient was guarding the knee in an extension position due to severe pain. The distal neurovascular examination was normal.

**Fig. 1:**
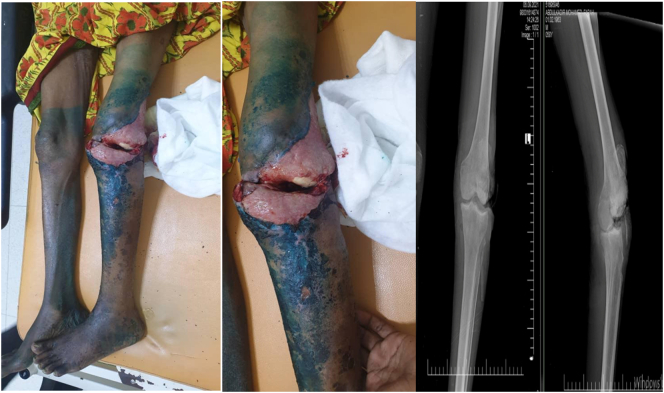
Preoperative clinical pictures and radiographs of the knee demonstrates reduced but severely infected anterior wound of the knee.

Radiographic examination revealed a reduced knee with a high patella and quadriceps tendon retraction, but no bone injury. Doppler ultrasound of the lower limbs revealed no vascular damage, there was no evidence of deep venous thrombosis (Fig. 1).

**Fig. 2:**
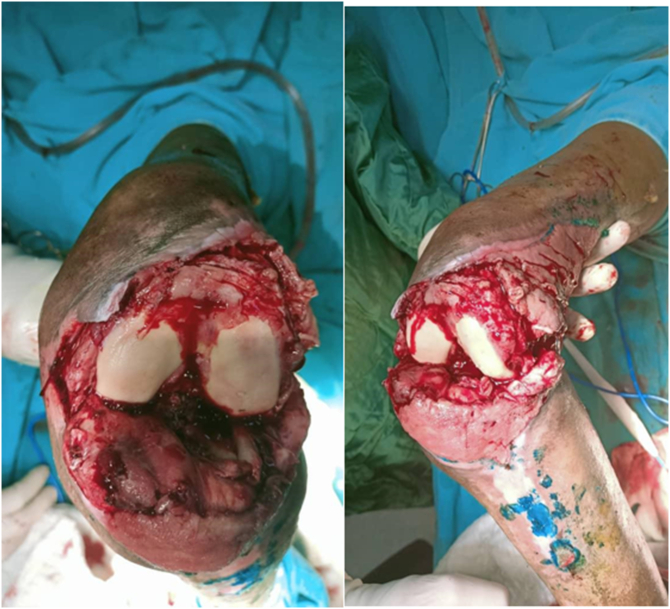
Intraoperative clinical image of the knee joint indicates instability, knee cartilage erosion, severely damaged joint ligaments, and a torn patellar tendon.

Under general anesthesia, the knee was unstable. Degenerative ruptures of the anterior cruciate and lateral collateral ligaments were noted. There were fatty degenerative remnants of the patellar tendon and joint capsule as well (Fig. 2). The wound surfaces were washed with 3% hydrogen peroxide and irrigated with considerable amounts of saline. Wound margins were cut until fresh bleeding appeared. Daily normal saline and povidone wound dressing was done, parenteral antibiotic therapy was administered for two weeks.

**Fig. 3:**
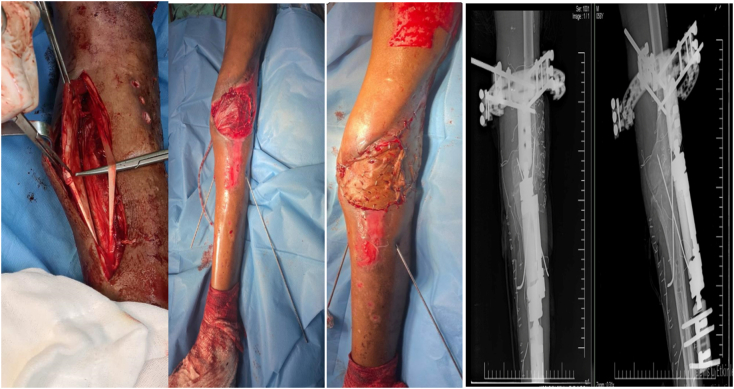
Intraoperative medial gastrocnemius flap elevated and transposed to reconstruct the defect. Full thickness skin graft used to cover muscular belly. Anteroposterior and lateral radiographs showing reduction of the joint and fixation with hybrid external fixator augmented by two crossed Steinmann pins.

Two weeks later wound culture yielded no bacterial growth but there was insufficient coverage of the knee and extensive erosion of the joint cartilages, decision was taken to perform medial gastrocnemius flap and knee arthrodesis. The entire lower limb was prepared and draped in a sterile fashion, the joint was debrided thoroughly to remove all necrotic and infected tissues, the joint surfaces were prepared using an oscillating saw to create flat surfaces and maximize bone contact for successful arthrodesis. A longitudinal incision was made along the medial belly of the gastrocnemius muscle, blunt dissection between the medial gastrocnemius belly and the soleus muscle was carried and the muscle belly was detached at its insertion at the Achilles tendon. A medial tunnel was created at the joint level to pass the gastrocnemius muscle belly so it can be laid over the anterior defect, interrupted absorbable 2-0 vicryl was used to secure the flap in place. Partial thickness skin graft harvested from the ipsilateral thigh was used to cover the muscle belly. The arthrodesis surfaces were fixed temporarily using two strong Kirschner-wires, preoperatively assembled hybrid external fixator was used to protect fixation with two pin rods in the femur and three pin rods into the tibia. Wounds were covered with furacin cream (Fig. 3). Postoperatively the patient was instructed to avoid dressings for the first five days after surgery and to limit weight bearing for 45 days. After two weeks of antibiotics, analgesics, and regular wound care, the patient's wound healed with no signs of infection.

**Fig. 4:**
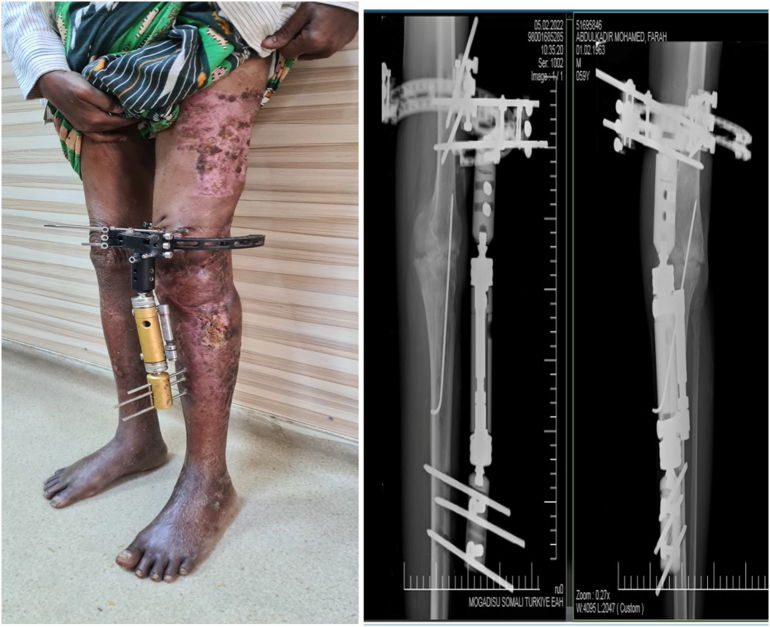
Three months clinical and radiographs follow-up of the knee showing good alignment and excellent healing of the flap without any signs of infection.

Three months of clinical and radiological follow-up demonstrated excellent healing of the flap and donor site, as well as promising arthrodesis union (Fig. 4).

Operator: Dr. Hassan Salad, Orthopedic, and Trauma Senior Resident at Mogadishu Somali Turkish Training and Research Hospital, Dr. Huseyin Taskoparan, Orthopedic and Trauma consultant. Dr. Duran Hashi, Anesthesia and Reanimation consultant. The operation was done at Mogadishu Somali Turkish Training and Research Hospital in Mogadishu, Somalia.

## 3 Clinical discussion

Knee dislocation and subluxation is a rare pathologic condition that can be accurately detected and treated during an emergency assessment [6], [7]. Most acute knee dislocations are either spontaneously reduced at the scene of the accident or reduced by clinicians in the emergency department. Missed diagnosis or treatment in the hands of traditional bone setters may result in chronic knee dislocation. Ilizarov external fixators, Steinmann pins, arthrodesis have been used to treat neglected chronic knee dislocation [8].

The combination of neglected open knee dislocation is an exceptionally rare injury whose incidence and prognosis are poorly characterized, and has been rarely reported in the English literature. These types of injuries not only necessitate the participation of an experienced orthopedic surgeon, but also that of plastic surgeons to cover the wound.

The most critical component of treating neglected open knee injuries is to successfully cover the knee and restore as much limb function as possible. Douglas G and his colleagues treated 14 open knee dislocations over an 18-year period, nine patients had concomitant neurovascular injuries that required immediate revascularization, eight patients had wound healing problems that were mostly treated with wound closure, and one patient had a gastrocnemius flap. Their final results included three above knee amputations, one knee fusion, and one total knee arthroplasty [1].

Vicente-Guillen et al. used two stage external fixation and arthrodesis to successfully treat a middle-aged woman with long-standing posterior dislocations of the knee [9]. Richard's expertise with chronic joint dislocations revealed that primary knee arthrodesis would be the best therapeutic option for neglected knee dislocation [10].

In the case described herein, the reason for delayed treatment was quite peculiar, as the patient was treated by traditional bonesetter in the acute setting which resulted in severe infection of the wound, fortunately salvage of the limb was possible after aggressive wound debridement, medial gastrocnemius flap and arthrodesis.

## 4 Conclusion

Delayed treatment of open knee dislocations is a relatively unusual injury that can be limb threatening. The use of aggressive soft tissue debridement and early antibiotic therapy, followed by a gastrocnemius flap and arthrodesis, has been demonstrated to be beneficial in the treatment of these injuries.

## Consent

Written informed consent was obtained from the patient for publication of this case report and accompanying images. A copy of the written consent is available for review by the Editor-in-Chief of this journal on request.

## Provenance and peer review

Not commissioned, externally peer-reviewed.

## Ethical approval

Ethical approval was not needed for writing a case report in our settings.

## Funding

The authors received no funding from any individual or institution, and this work is completely voluntary.

## Guarantor

Hassan Salad Ibrahim.

## Research registration number

Not applicable.

## CRediT authorship contribution statement

Hassan Salad Ibrahim was involved in study design, data acquisition, drafting the article, revising it critically, and finally approved the manuscript.

## Declaration of competing interest

The authors report no conflict of interest of any sort.
